# Murine gammaherpesvirus M2 antigen modulates splenic B cell activation and terminal differentiation in vivo

**DOI:** 10.1371/journal.ppat.1006543

**Published:** 2017-08-02

**Authors:** Shariya Terrell, Samuel H. Speck

**Affiliations:** 1 Department of Microbiology & Immunology, Emory University School of Medicine, Atlanta, GA, United States of America; 2 Emory Vaccine Center, Emory University School of Medicine, Atlanta, GA, United States of America; University of Pennsylvania Medical School, UNITED STATES

## Abstract

Murine gammaherpesvirus 68 (MHV68) infection of laboratory strains of mice has provided a tractable small animal model for dissecting gammaherpesvirus pathogenesis. The MHV68 latency associated antigen M2 promotes viral latency establishment in germinal center (GC) B cells and plays an important role in virus infection of plasma cells (PCs), which is linked to virus reactivation. More recently, M2 has been highlighted as a potent immunomodulatory molecule capable of hindering both cell-mediated and humoral immunity to MHV68 infection and subsequent challenges. M2 expression in B cells results in activation of B cell receptor signaling pathways that promote proliferation, differentiation, and cytokine production—a hallmark of gammaherpesviruses. In this study, we utilized an adoptive transfer model to explore the biological consequence of M2 expression in activated B cells in vivo. Secondly, we engineered and validated two independent MHV68 M2 reporter viruses that track M2 protein expression in latently infected B cells during infection. Here we demonstrate that upon adoptive transfer into naive mice, M2 expression promotes activated primary B cells to competitively establish residency in the spleen as either a GC B cell or a PC, most notably in the absence of an ongoing GC reaction. Moreover, M2 antigen drives robust PC differentiation and IL10 production in vivo in the absence of other viral factors. Lastly, we confirm that M2 expression during MHV68 infection is localized to the GC compartment, which is a long term latency reservoir for gammaherpesviruses. Overall, these observations are consistent with, and extend upon previous reports of M2 function in B cells and within the context of MHV68 infection. Moreover, this work provides support for a model by which M2-driven dysregulation of B cell function compromises multiple aspects of antiviral immunity to achieve persistence within the infected host.

## Author summary

Gammaherpesvirus (GHVs), which primarily infect B cells, are capable of exploiting B cell biology to achieve a stable and persistent infection for the lifetime of the host. GHV infections traffick to germinal center (GC) B cells and plasma cells (PCs), which are important immune effectors that promote the generation of protective antibodies in response to pathogens. The mechanism by which murine gammaherpesvirus 68 (MHV68) M2 latency protein activates B cell receptor signaling pathways to modulate the immune response to infection and further promote viral pathogenesis within the GC B cell and PC compartments is not completely understood. Here we demonstrate that M2 expression alone, in the absence of other viral factors, drives robust PC differentiation and IL10 production in vivo. Moreover, M2 promotes the accumulation of splenic GC B cells, which was subsequently verified as the site for potent M2 expression during latent MHV68 infection. Our work further substantiates a model in which a viral protein dysregulates B cell activation, differentiation, and cytokine production to create a permissive environment for viral persistence in the infected host. This work justifies further investigations addressing the impact of GHV latency antigen function within the GC reaction and overall host response to infection.

## Introduction

Herpesvirus infections characteristically exhibit dynamic host-pathogen interactions that promote viral persistence for the lifetime of the infected host (reviewed in [1]). Gammaherpesviruses (GHVs) primarily infect and establish latency in B cells and can potentially trigger lymphomagenesis in an immunosuppressive environment. For example the human GHVs, Epstein-Barr virus (EBV) and Kaposi’s sarcoma-associated herpesvirus (KSHV), have been identified as the etiological agents of Burkitt’s lymphoma and Kaposi’s sarcoma, respectively [2, 3]. Although studies utilizing immortalized latently infected cells lines and transgenic mice have provided valuable insights into the functions GHV antigens in B cells, the narrow host cell tropism of EBV and KSHV, coupled with the lack of robust small animal models for these human pathogens, has significantly impacted research efforts with respect to viral pathogenesis studies in the infected host. Murine gammaherpesvirus 68 (MHV68), which exhibits similar genomic organization and extensive sequence homology with other GHVs, is a natural rodent pathogen that has proven to be a useful tool for studying latency, reactivation, and pathogenesis [4]. MHV68 infection of laboratory strains of mice results in a brief phase of acute replication followed by subsequent latency establishment in macrophages, dendritic cells and B cells, with the latter representing the predominant latency reservoir in vivo [5–7]. Combined with the fact that MHV68 can infect various cell lines in vitro, this model provides a robust system that can be utilized to interrogate the functional role of both host and viral factors in GHV pathogenesis.

Non-specific B cell activation and lymphoproliferation are markers commonly associated with herpesvirus infections and this phenomenon is further exploited by GHVs that encode unique latency associated antigens capable of modulating B cell signaling activity [8–10]. EBV proteins LMP1 and LMP2a are constitutive CD40 and BCR mimics, respectively, that provide latently infected B cells with survival signals in the absence of T cell help and antigen recognition [11, 12]. Transgenic expression of LMP1 or LMP2a in murine B cells results in enhanced survival, proliferation, differentiation, and immunoglobulin production [11–13]. During latent EBV infection, LMP1 and LMP2a expression is associated with naïve and germinal center (GC) B cells and their proposed function is to drive latently infected B cells through the GC reaction and into the long lived memory B cell reservoir [14, 15]. However, the ultimate impact of GHV latency antigen signaling activity with respect to B cell differentiation and viral trafficking during EBV infection is currently under debate. The MHV68 M2 latency associated antigen, although it has no obvious viral or cellular homologs, exhibits similar functions upon expression in murine B cells. M2 functions as molecular scaffold that triggers the activation of BCR signaling pathways resulting in B cell proliferation, differentiation, and immunoglobulin production in vitro [16–19]. During MHV68 infection in vivo, M2 expression enhances latency establishment in splenic GC B cells and is particularly critical for plasma cell (PC) differentiation of latently infected B cells and subsequent virus reactivation, which is a hallmark of GHV infection [20–24].

Recently, an expanding body of literature provides convincing evidence that B cells exhibit potent immunoregulatory activity via provision of IL10 in a number of auto-immune diseases and pathogen infections [25–30]. For example, B-cell derived IL10 suppressed pathogen-specific CD4+ T cell, natural killer, and neutrophil responses which correlated with decreased survival in a salmonella infection model [28]. Activation of TLR, CD40, and/or BCR signaling induces IL10 production in B cells, but the mechanisms underlying the development and function of IL10-competent B cells have yet to be fully elucidated [26, 31, 32]. The MHV68 M2 antigen drives robust IL10 expression from B cells through upregulated expression of B cell differentiation factor IRF4, which supports B cell proliferation and differentiation in vitro [17, 19]. Accordingly, M2 expression during MHV68 infection contributes to elevated IL10 serum levels, reduced antiviral CD8+ T cell activity and attenuated MHV68-specific humoral responses [19, 33]. Moreover, loss of M2 expression restored parasite-specific antibody responses that effectively rescued animals from a lethal co-infection with MHV68 and rodent plasmodium [33]. Therefore, further investigation of M2 may provide important insights into common mechanisms by which GHVs, as well as potentially other pathogens, manipulate B cell biology to alter the host response to infection.

While previous studies have reported the detection of M2 transcripts in various splenic B cell subsets following MHV68 infection, the mechanism by which M2 antigen expression dampens antiviral immunity and promotes MHV68 pathogenesis in vivo has not been rigorously addressed [5, 34, 35]. In this study, we sought to interrogate: (i) the impact of M2 expression in the context of adoptively-transferred splenic B cells; and (ii) the location of M2 antigen expression during latent MHV68 infection in vivo. Here, we show that M2 expression alone, in the absence of other viral factors, dramatically modulates B cell activity similar to that observed during MHV68 infection in mice. Moreover, these observations may represent a common mechanism by which MHV68 and other pathogens subvert the adaptive immune response by dysregulating B cell function during infection.

## Results

### Validation of M2 reporter constructs that track M2 expression in splenic B cells

**Fig 1:**
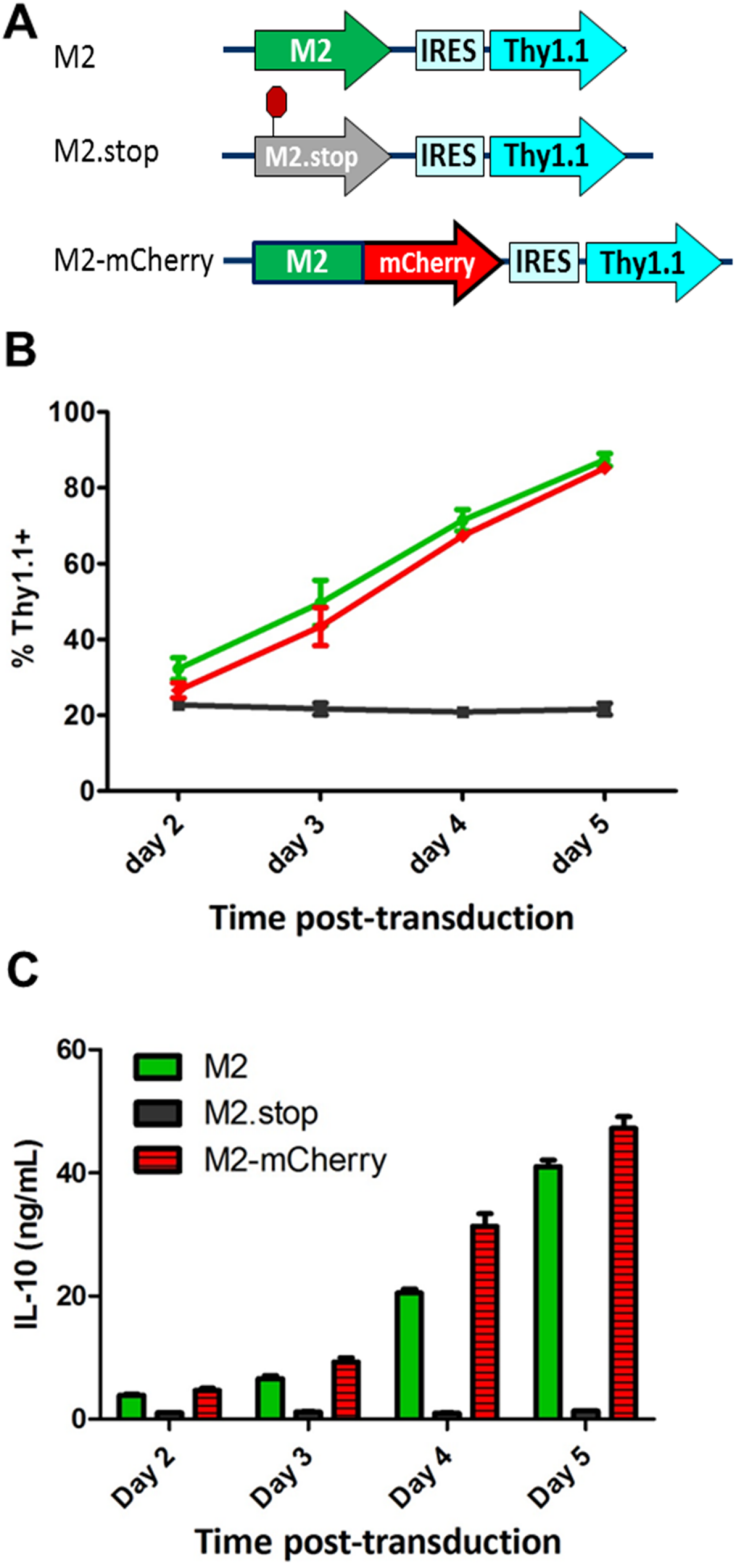
M2-mCherry reporter construct recapitulates IL10-dependent expansion of M2-transduced primary B cells in vitro. (A) Retroviruses were derived from replication defective murine stem cell virus (MSCV) vectors that drive constitutive expression of the wild type M2, M2.stop (M2 ORF containing a translational stop codon at amino acid 13), or M2-mCherry upstream of the IRES-Thy1.1 cassette. (B) Primary B cells were isolated from naïve mice by negative selection and stimulated with LPS prior to retroviral transduction. Cell cultures were harvested at the indicated time points and cell surface Thy1.1 expression was analyzed by flow cytometry. (C) Supernatants from retrovirally transduced B cell cultures were analyzed for IL10 secretion by ELISA. Mean and standard deviation values for (B) and (C) were derived from compiling data from two independent experiments containing three replicates per condition per time point.

Extensive characterization of MHV68 M2 antigen function in vitro has revealed a wealth of information, namely that it functions as an adaptor protein by triggering the assembly of multimeric protein complexes that activate various B cell signaling pathways [16–18, 36]. In particular, retroviral transduction of LPS-stimulated primary B cells with a constitutively active M2 expression vector results in enhanced B cell proliferation and IL10 production [19]. Due to the fact that IL10-dependent cell proliferation requires a functional M2, we utilized this retroviral transduction system as a screening tool to evaluate the detection efficacy of an intracellular marker that could monitor M2 protein expression without adversely disrupting its function. To this end, an M2-mCherry transgene was generated by fusing the M2 ORF upstream of the mCherry fluorescent protein sequence with an intervening 30 amino acid F2A peptide derived from the foot-and-mouth disease virus 2A [37]. The incorporation of the F2A peptide allows for cotranslation of separate polypeptides from a single mRNA transcript [37], thus enhancing the likelihood of preserving both M2 and mCherry functionality. The M2-mCherry construct was cloned into a replication-defective murine stem cell virus (MSCV) vector upstream of an IRES-Thy1.1 cassette, which facilitates detection of transduced B cells by surface Thy1.1 expression (Fig 1A) [19]. Primary B cells were isolated from bulk splenocytes by negative selection and stimulated overnight with LPS prior to transduction with the MSCV-M2-mCherry retrovirus, or the previously described wild type MSCV-M2 and negative control MSCV-M2.stop retroviruses (the latter contains a translation stop codon at amino acid 13 of the M2 open reading frame) (Fig 1A). Following retroviral transduction, B cell cultures were harvested in triplicate at each time point and monitored for cell surface Thy1.1 expression and IL10 production. Consistent with previously published reports [17, 19], transduced cells (Thy1.1+) expressing M2 expanded to ~90% of the culture and secreted ~40ng/mL of IL10 by day five post-transduction, which was not observed in M2.stop-transduced cultures (Fig 1B and 1C). M2-mCherry expressing cultures exhibited Thy1.1+ B cell expansion and robust IL10 production nearly identical to that observed with the wild type M2 expression construct (Fig 1B and 1C), indicating that M2 protein expressed from the fusion gene possessed wild type M2 function in B cells.

**Fig 2:**
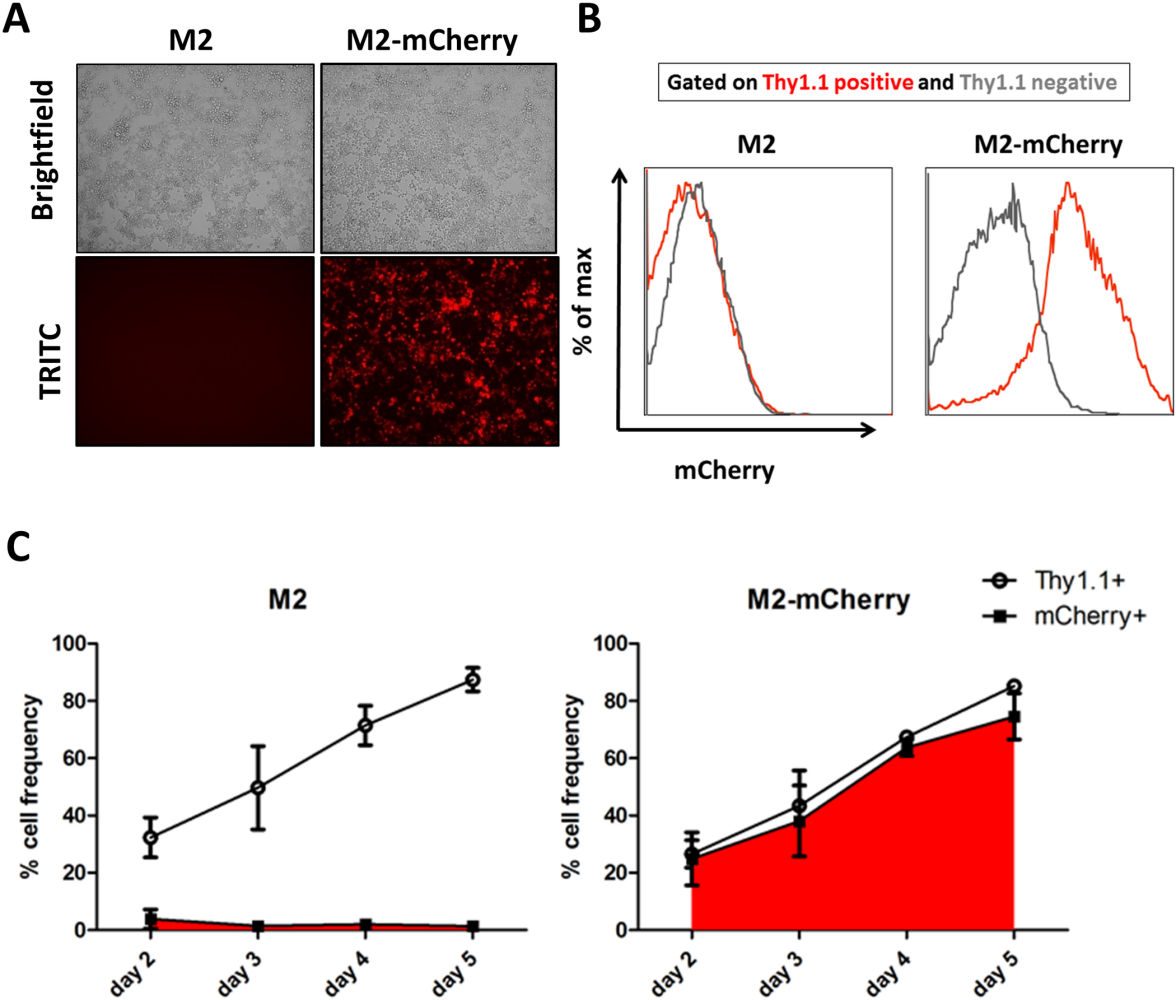
Detection of M2 reporter activity in activated splenic B cells. (A) Representative images of transduced primary B cells obtained from brightfield and TRITC filters at day 3 post-transduction. (B) Representative flow cytometry histograms obtained at day 3 post-transduction compare mCherry expression in Thy1.1 positive (red) and Thy1.1 negative (grey) populations as determined by FACS analysis. (C) Frequencies of cell surface Thy1.1 and intracellular mCherry expression of M2-transduced (left panel) and M2-mCherry-transduced (right panel) B cell cultures were monitored over the time course by flow cytometry. Mean and standard deviation values were derived from compiling data from two independent experiments containing three replicates per condition per time point.

Following verification of M2-mCherry function in primary B cells, we sought to evaluate the efficiency of mCherry detection with respect to cell surface marker Thy1.1, which serves as a marker for transduction efficiency [19]. We initially visualized robust mCherry expression by fluorescence microcopy in transduced primary B cell cultures expressing M2-mCherry, but not in cells expressing wild type M2 (Fig 2A). Analysis of intracellular and cell surface marker expression by flow cytometry demonstrated that the MSCV-M2-mCherry transduced (Thy1.1+) population exhibited robust mCherry expression, which was not observed in untransduced (Thy1.1-) or MSCV-M2 transduced B cells (Fig 2B). Accordingly, mCherry and Thy1.1 expression were detected at a 1:1 ratio in M2-mCherry-transduced B cells throughout the time course, while the frequency of mCherry+ population in M2-transduced B cell cultures remained at ≤5% of total cells (Fig 2C). Thus, we concluded that both M2-mCherry and M2-Thy1.1 constructs could serve as faithful reporters that preserve wild type M2 function and facilitate detection of M2 protein expression in murine B cells.

### M2 expression promotes differentiation of activated splenic B cells in naïve mice

**Fig 3:**
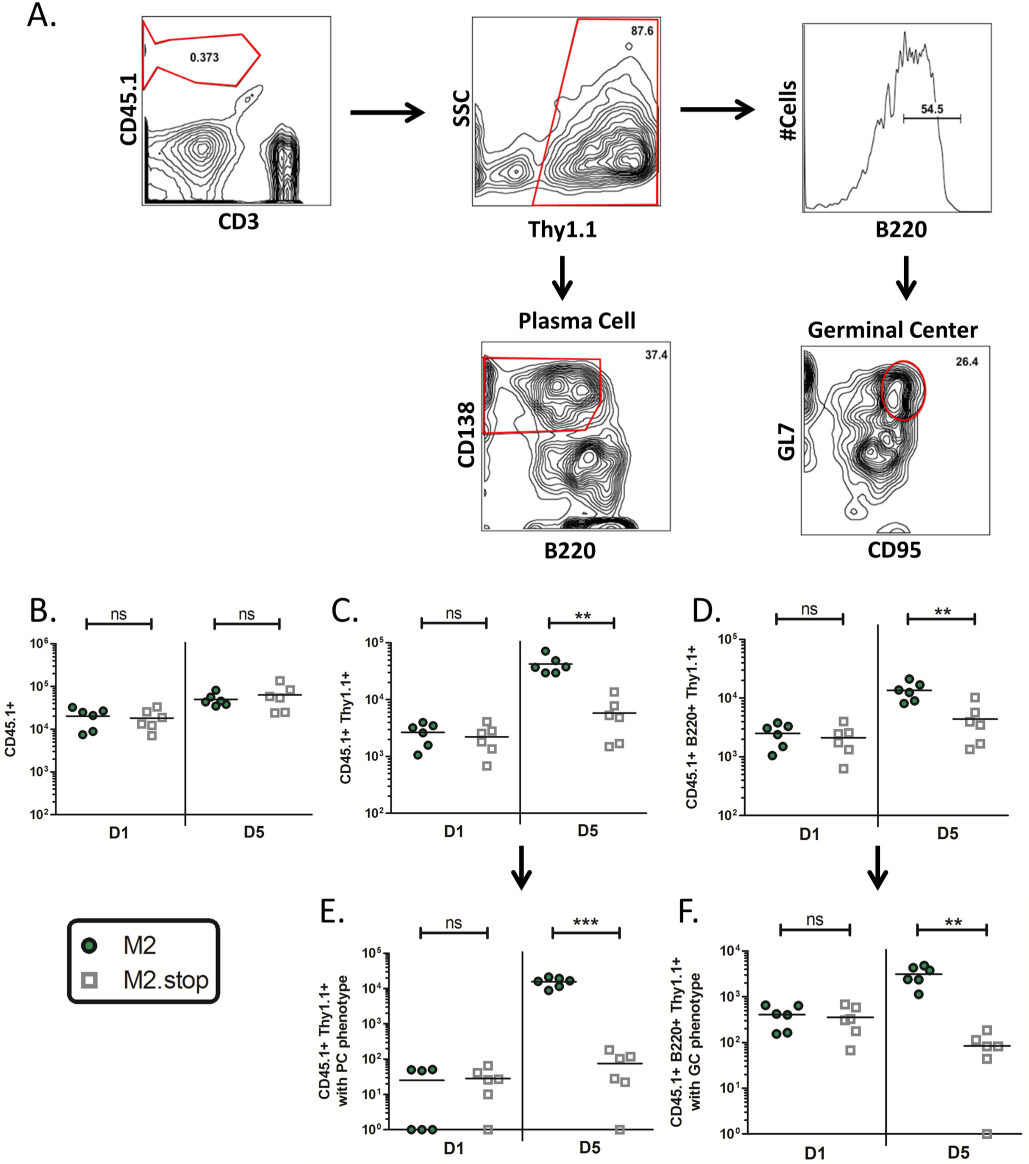
M2-transduced B cells establish splenic plasma cell and germinal center B cell populations upon adoptive transfer in naïve mice. Primary splenic B cells isolated by negative selection from naïve CD45.1+ donors were stimulated with LPS and subsequently transduced with M2- or M2.stop- retroviruses prior to adoptive transfer into naïve C57BL/6 mice (CD45.2+). Splenocytes from adoptive transfer recipients were harvested at D1 and D5 post transfer and cell surface markers were analyzed by flow cytometry. Data was compiled from two independent experiments with 3 mice/group. Each data point represents one animal and the horizontal bar represents the mean. Statistics were determined by two-tailed unpaired t test with Welch’s correction. (A) Representative flow plots depicting gating strategies to identify and subphenotype the transduced adoptively transferred B cell population (CD45.1+Thy1.1+) for plasma cell (B220^Lo^CD138^Hi^) or germinal center B cell (B220+GL7^Hi^CD95^Hi^) cell surface markers. Absolute numbers of splenic CD45.1+ B cells (B), CD45.1+Thy1.1+ B cells(C), CD45.1+B220+Thy1.1+ B cells (D), CD45.1+Thy1.1+ B cells displaying a plasma cell phenotype, and (E) CD45.1+B220+Thy1.1+ B cells displaying a germinal center phenotype (F).

Constitutive M2 expression in B cells results in upregulated expression of several plasma cell differentiation factors leading to robust IRF4-driven IL10 production, immunoglobulin secretion, and differentiation into an activated pre-plasma memory B cell (CD19^+^GL7^Hi^B220^Lo^sIgD^-^sIgG^+^CD138^Lo^) in vitro [17, 19, 22]. To further evaluate the consequence of M2-driven activation of B cell signaling pathways, we sought to determine the fate of M2-expressing B cells upon adoptive transfer into a naïve host. Primary splenic B cells isolated from CD45.1+ donors were stimulated with LPS and retrovirally transduced with MSCV-M2 or MSCV-M2.stop retroviruses as previously described, prior to adoptive transfer into the peritoneum of naïve C57BL/6 mice (CD45.2+). Splenocytes from adoptive transfer recipients were harvested at one and five days post-transfer and analyzed by flow cytometry to derive the absolute number of cells per spleen for the indicated populations (Fig 3A). A fraction of the adoptively transferred B cell population trafficked to the spleen and the overall recovery of CD45.1+ cells from both M2 and M2.stop adoptive transfer recipients was comparable throughout the time course (Fig 3B). However, transduced B cell numbers (CD45.1+Thy1.1+) were 7-fold higher in M2 animals as compared to M2.stop animals at D5 post transfer (Fig 3C). Interestingly, M2-transduced B cells comprised ≥80% of the total splenic CD45.1+ population at D5 post transfer while the frequency of M2.stop-transduced B cells remained at just ~10%. We also observed a modest yet significant 3-fold increase in M2-transduced B cells numbers in the B220+ fraction versus the corresponding M2.stop-transduced population at D5 post transfer (Fig 3D). Due to the dynamic nature of this system, we are unable to address whether M2 expression confers enhanced survival or proliferation—although a previous study demonstrated that both factors contribute to the expansion of M2-transduced B cells in vitro [19]. Next, we sought to evaluate whether the splenic microenvironment could further influence the differentiation state of M2-expressing B cells in vivo. Transduced B cells from the total CD45.1+ B cell population and the CD45.1+B220+ fraction were subphenotyped for cell surface markers consistent with plasma cell (PC) and germinal center (GC) B cell phenotypes, respectively (Fig 3A). The numbers of transduced B cells exhibiting either a PC (B220^Lo^CD138^Hi^) or GC (B220+GL7^Hi^CD95^Hi^) phenotype per spleen at D1 post transfer were indistinguishable for both conditions (Fig 3E and 3F). However, by D5 post transfer, mice receiving M2-transduced B cells exhibited a striking 208-fold and 37-fold enhancement of Thy1.1+ B cells exhibiting a PC or GC phenotype, respectively, versus the corresponding populations in mice receiving M2.stop-transduced B cells. Importantly, the observed decline in M2.stop transduced B cells exhibiting a GC phenotype from D1 to D5 post-transfer cannot be attributed to a massive loss of M2.stop-transduced B cells, as we detected a substantial Thy1.1+ population in M2.stop adoptive transfer recipients at D5 post-transfer (Fig 3C). These results clearly demonstrate that PC and GC B cell populations expressing M2 exhibit a competitive advantage over the untransduced B cell population with respect to maintaining residence in the spleen.

**Fig 4:**
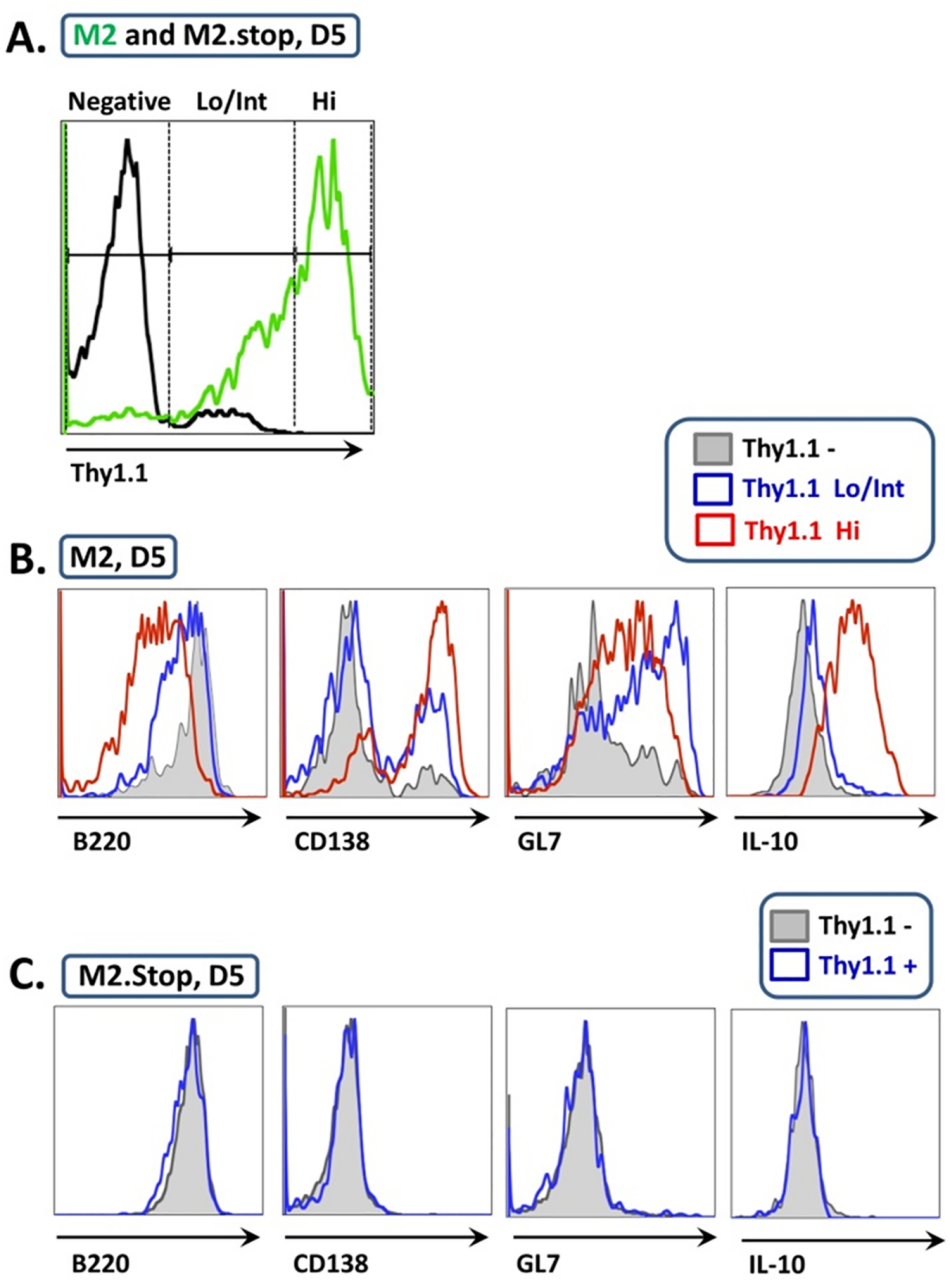
M2 antigen expression modulates splenic B cell activation and differentiation in vivo. The transduced adoptively transferred B cell populations from M2 and M2.stop animals were analyzed for cell surface marker expression as a function of transgene expression. Data is representative of two independent experiments with 3 mice/group. A) Representative histogram depicting Thy1.1 expression within the adoptively transferred (CD45.1+) population in M2 and M2.stop animals at D5 post transfer. Thy1.1 expression in the CD45.1+ fraction was divided into Thy1.1 –, Thy1.1 Lo/Int (or Thy1.1 +), and Thy1.1 Hi populations. Representative histograms displaying B cell surface marker expression and intracellular IL10 production corresponding to Thy1.1 expression intensities are displayed for M2 (B) and M2.stop (C) transduced B cells.

To further evaluate B cell differentiation status as a function of M2 concentration, adoptively transferred B cells were divided into Thy1.1 –(no M2 expression), Thy1.1 Lo/Int (low to intermediate levels of M2 expression), and Thy1.1 Hi (high level M2 expression) populations (Fig 4A), followed by analysis of cell surface marker expression for each group. Similar to previous reports, B220 expression was significantly downregulated in the M2-Thy1.1 Hi population, while M2.stop-Thy1.1+ B cells exhibited levels of B220 similar to that of the untransduced Thy1.1 –population ([17, 19]; Fig 4B and 4C). Consistent with M2-driven IRF4 expression in B cells [17], the PC marker CD138 was robustly upregulated in ca. 70% of transduced B cells expressing the highest levels of M2 (Thy1.1 Hi) and ca. 15% of B cells expressing intermediate or low levels of M2 (Thy1.1 Lo/Int), as compared to ca. 2% of the untransduced B cell population (Fig 4B). We also observed graded expression of the B cell activation marker GL7, which appeared to be highest in the M2-Thy1.1 Lo/Int population and was almost undetectable in M2.stop Thy1.1+ B cells (Fig 4). Finally, we assessed intracellular IL10 protein expression in these transduced B cell populations–which revealed robust induction of IL10 in the M2 Thy1.1 Hi population and a more modest induction of IL10 expression in the Thy1.1 lo/Int B cell population (Fig 4B). This is consistent with our previous studies demonstrating that IL10 is a downstream target of M2-induced IRF4 expression [17]. Overall, this data confirms that the observed changes in cell surface marker expression are a direct consequence of M2 expression–as opposed to the mere ability to survive within the splenic microenvironment longer than the M2.stop transduced B cell populations–and further establishes M2 as a potent factor with respect to B cell activation and differentiation. In conclusion, this is the first evidence that M2 expression alone, in the absence of an ongoing infection and other viral factors, can support the activated GC B cell phenotype and directly promote PC differentiation and IL10 production from stimulated B cells in vivo.

### Recombinant MHV68-H2bYFP M2 reporter viruses efficiently establish latency and reactivate infectious virus from splenic B cells

**Fig 5:**
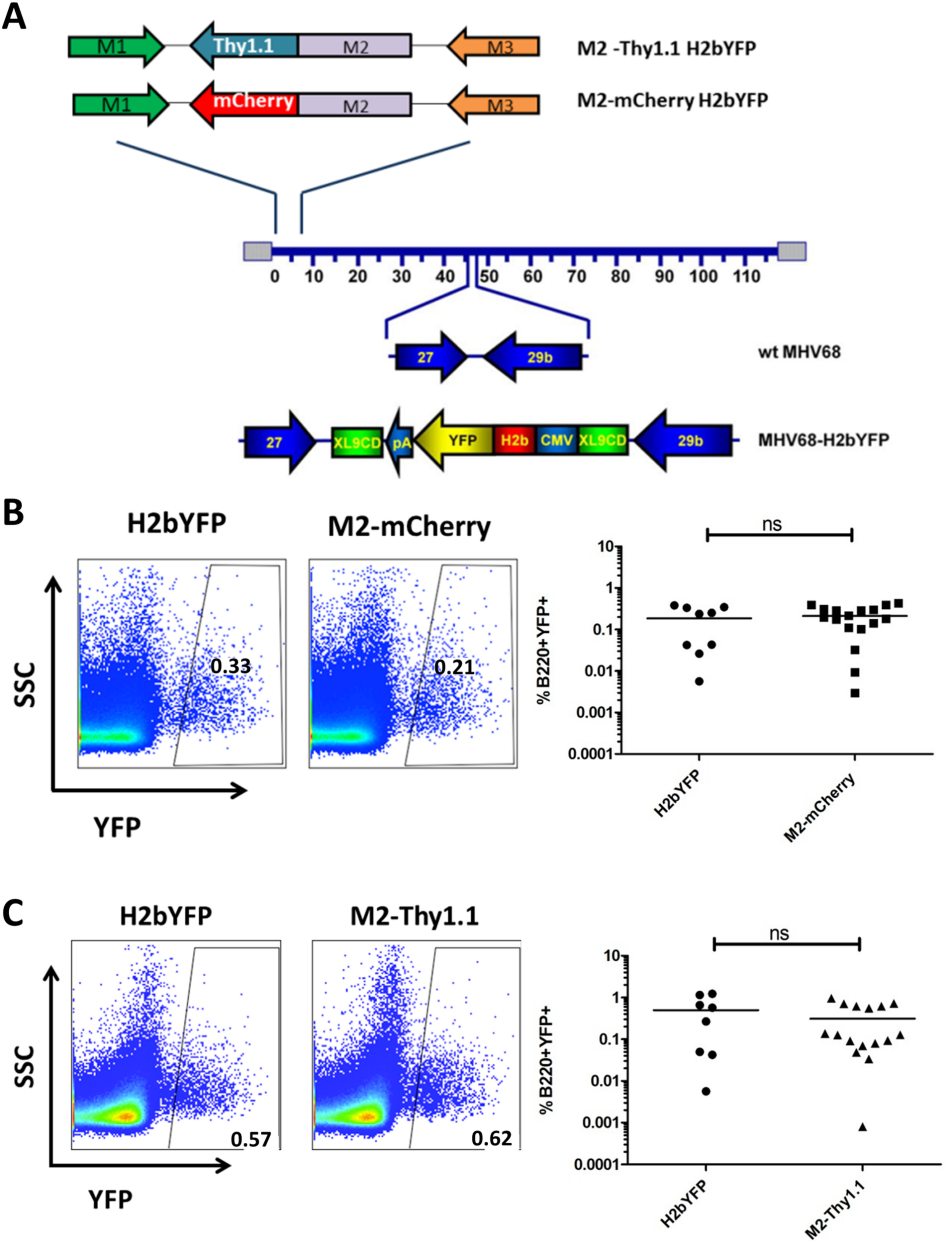
Generation of recombinant MHV68 M2 reporter viruses. (A) The M2-Thy1.1 or M2-mCherry reporter construct (Fig 1A) was incorporated at the M2 locus in the MHV68 H2bYFP background under the control of the native M2 promoter. C57BL/6 mice were infected at 1000PFU via the intraperitoneal route and splenocytes were harvested at 14 dpi. Statistics were determined by two-tailed unpaired t test with Welch’s correction. (B and C) Representative flow plots (left) and quantitation (right) of B220^+^YFP^+^ frequency in latently infected splenocytes were obtained from infections comparing the parental MHV68 H2bYFP virus and two (2) independent clones of either the MHV68 H2bYFP M2-mCherry (B) or M2-Thy1.1 (C) virus. Each data point represents one animal and the horizontal bar represents the mean.

Given that the phenotype of adoptively transferred M2-transduced B cells was consistent with MHV68 latency reservoirs in vivo, we sought to interrogate the context of M2 expression during infection. To this end, we generated two independent M2 reporter viruses in the background of the previously described MHV68-H2bYFP virus, which marks latently infected B cells with intracellular YFP that can be detected by flow cytometry [38]. Previous studies demonstrated that YFP+ splenic B cells contain the viral genome and are capable of reactivating infectious virus, which allows us to utilize YFP expression as an internal control for latency establishment efficiency. To track M2 expression under the control of its native promoter, the M2 ORF in the context of the MHV68-H2bYFP BAC genome was substituted with either the M2-mCherry or the M2-Thy1.1 reporter construct sequences via the galK recombination system (Fig 5A) [39]. The recombinant MHV68-H2bYFP BAC clones were sequenced at the M2 locus to confirm the presence of the inserted constructs and the integrity of the viral genome was evaluated by RFLP analysis.

**Fig 6:**
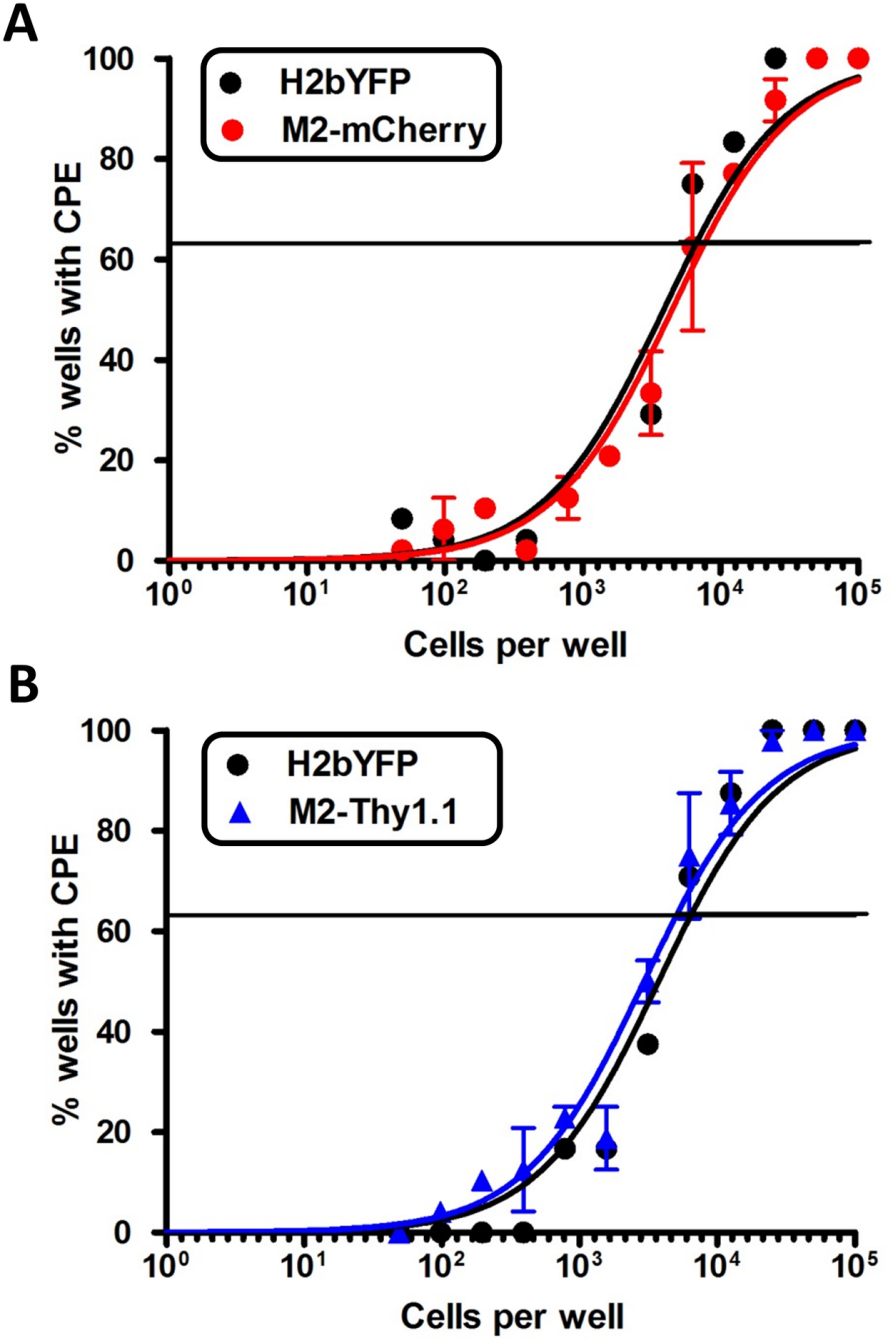
M2 reporter viruses efficiently reactivate virus from latently infected splenocytes ex vivo. Mice were infected at 1000PFU IP with the parental MHV68 H2bYFP virus and two independent clones of either the MHV68 H2bYFP M2-mCherry (A) or M2-Thy1.1 (B) viruses. Infected splenocytes harvested at 14 dpi were pooled from 3–5 mice/condition and serial dilutions were plated onto feeder cells as described in materials and methods. Cytopathic effect (CPE) was scored at 14–21 days post-explant to determine the frequency of cells that are capable of reactivation infectious virus ex vivo.

M2 expression during MHV68 infection in vivo enhances latency establishment and virus reactivation in a dose and route dependent manner [20, 21]. To evaluate overall viral fitness and account for any deleterious genomic alterations, we sought to quantify latency establishment efficiencies for the recombinant MHV68-H2bYFP M2 reporter viruses. C57BL/6 mice were infected at 1000 PFU via the intraperitoneal (IP) route with the parental MHV68-H2bYFP virus and two independent clones of either the M2-mCherry or M2-Thy1.1 reporter viruses and intracellular YFP expression in latently infected splenocytes was detected by flow cytometry. Consistent with previous reports, the H2bYFP virus displayed a typical variation in the frequency of latently infected B cells (%B220^+^YFP^+^) that averaged ~0.3% at 14dpi (Fig 5B and 5C) [40, 41]. M2-mCherry- and M2-Thy1.1- infected animals exhibited on average ~0.2% and ~0.3% B220^+^YFP^+^ cells, respectively, which was not significantly different to that of the parental MHV68-H2bYFP virus (Fig 5B and 5C). We subsequently evaluated virus reactivation from latently infected splenocytes upon explant into tissue culture by utilizing the previously described limiting dilution ex vivo reactivation assay [42]. The average frequency of cells reactivating infectious virus following M2-mCherry (1 in 6,309 cells) and M2-Thy1.1 (1 in 5,128 cells) infection was similar to that of parental H2bYFP (on average 1 in 6,165 cells) (Fig 6A and 6B). In conclusion, our extensive evaluation of the recombinant MHV68-H2bYFP M2 reporter viruses verify that the addition of exogenous sequences at the M2 locus did not dramatically alter M2-driven latency establishment in vivo and subsequent ex vivo virus reactivation from latently infected splenic B cells.

### M2 reporter activity is localized to GC B cells during latent MHV68 infection

**Fig 7:**
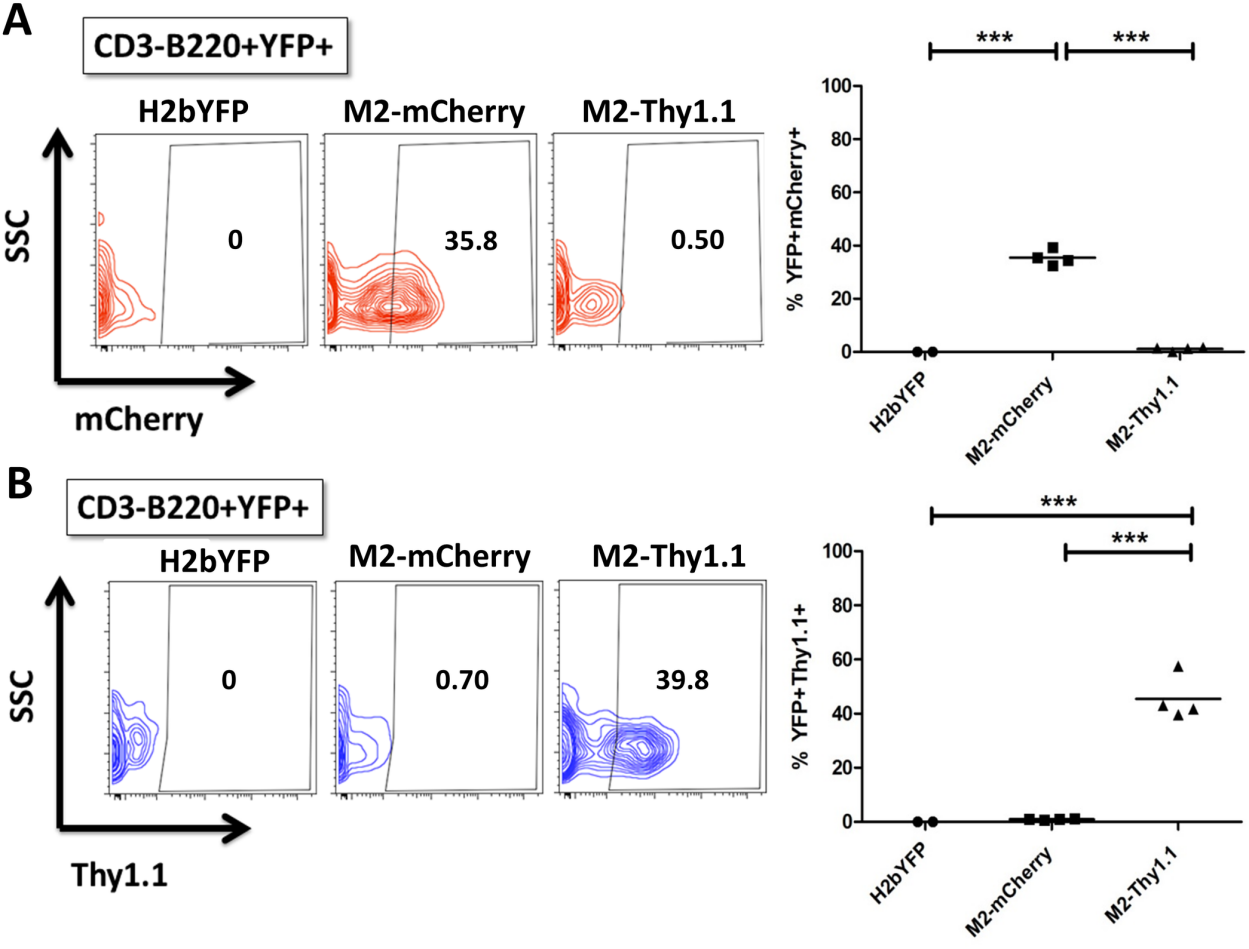
Robust M2 reporter expression is observed in a fraction of latently infected B cells in vivo. Mice were infected at 1000PFU IP with MHV68 H2bYFP and second generation MHV68 H2bYFP M2 reporter viruses M2-mCherry and M2-Thy1.1. Splenocytes were harvested at 14 dpi and analyzed for cell surface marker and fluorescent protein expression by flow cytometry. Each data point represents one animal and the horizontal bar represents the mean. Statistics were determined by one way analysis of variance (ANOVA) followed by Bonferonni’s multiple comparisons post tests. (A and B) Representative flow plots and quantitation of intracellular mCherry (A) or cell surface Thy1.1 (B) detection within the latently infected B cell compartment (CD3^-^B220^+^YFP^+^) of the indicated virus-infected animals.

**Fig 8:**
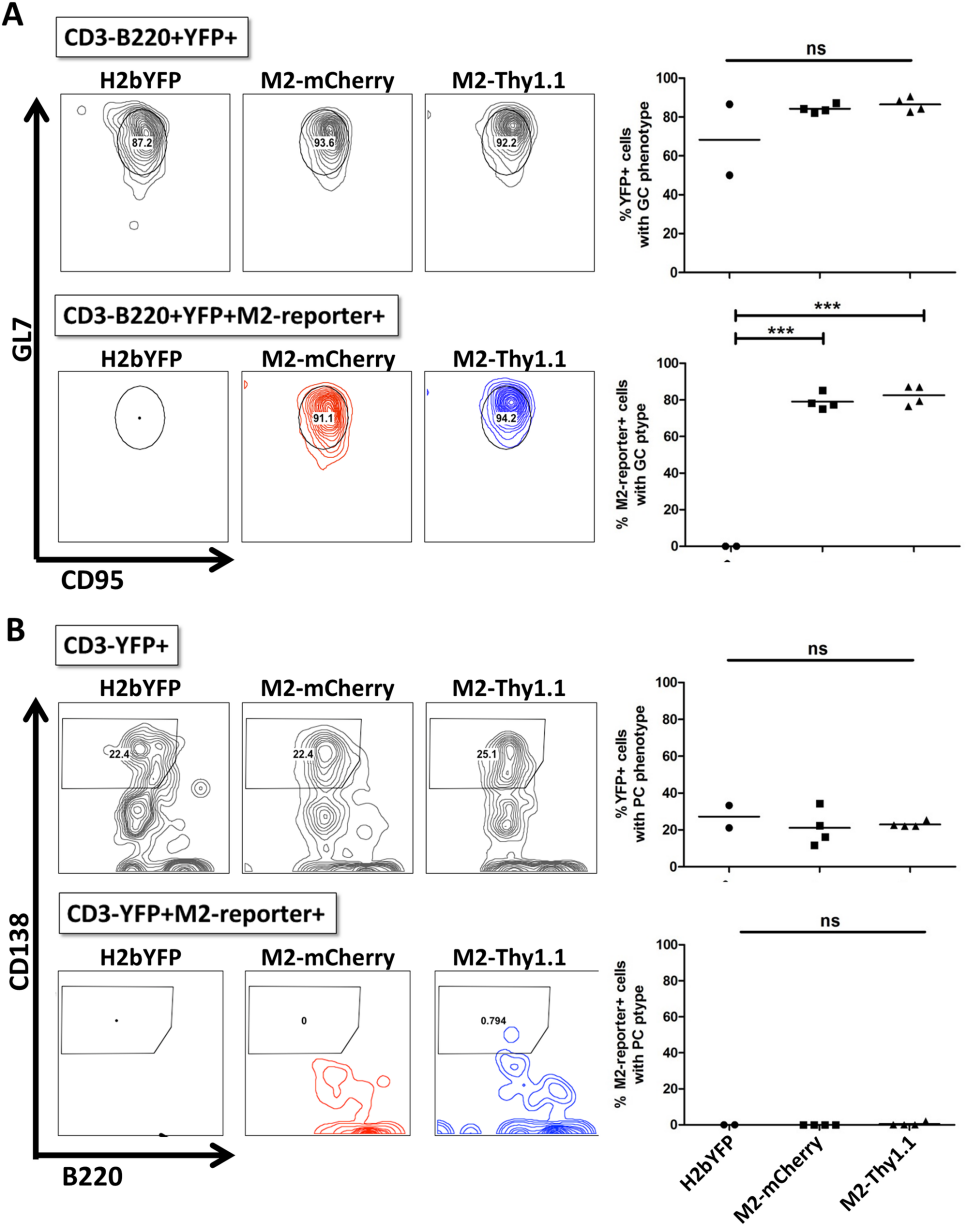
M2 protein is detected in the germinal center but not in the plasma cell compartment during latency. Mice were infected at 1000PFU via the intraperitoneal route with the parental MHV68 H2bYFP and second generation MHV68 H2bYFP M2 reporter viruses. Splenocytes were harvested and analyzed by flow cytometry at 14 dpi. Each data point represents one animal and the horizontal bar represents the mean. Statistics were determined by one way analysis of variance (ANOVA) followed by Bonferonni’s multiple comparisons post tests. (A) Representative flow plots (left) and quantitation (right) of latently infected B220^+^YFP^+^ population (top panel) and B220^+^YFP^+^M2-reporter^+^ cells (bottom panel) exhibiting a germinal center phenotype (GL7^Hi^CD95^Hi^). (B) Representative flow plots (left) and quantitation (right) of YFP+ (top panel) and YFP^+^M2-reporter^+^ cells (bottom panel) exhibiting a plasma cell phenotype (B220^Lo^CD138^Hi^).

Previous studies characterizing the MHV68-H2bYFP virus demonstrated that a majority of latently infected B cells faithfully display either a GC or a PC phenotype [38]. Incorporation of the M2.stop mutation in the MHV68-H2bYFP background revealed that M2 expression was dispensable for access to the GC B cell compartment, but critical for viral entry into the PC compartment that serves as a major reservoir for reactivating virus [22]. Accordingly, we sought to analyze the cell surface phenotype of M2-expressing B cells during latency establishment in vivo. To facilitate a side by side comparison of the engineered MHV68-H2bYFP M2 reporter viruses, animals were infected at 1000 PFU IP with either the parental H2bYFP virus, M2-mCherry virus, or M2-Thy1.1 virus. First, we quantified the M2 reporter positive population within the latently infected B220^+^YFP^+^ population by utilizing the parental H2bYFP virus as a negative control. Following IP infection, we detected a robust population of cells that was positive for intracellular mCherry or cell surface Thy1.1 expression with the respective reporter virus, which was not present in H2bYFP-infected animals (Fig 7). Very similar results were obtained with M2-mCherry and M2-Thy1.1 reporter virus infection, which resulted in ~40% of the latently infected B cell population exhibiting detectable M2 reporter expression at 14 dpi (Fig 7). Latently infected B220^+^YFP^+^ B cells characteristically exhibit a GC phenotype during latent MHV68 H2bYFP infection, which was recapitulated in M2 reporter virus infected animals (Fig 8A, upper panels). Accordingly, ≥90% of mCherry+ and Thy1.1+ cells within the B220^+^YFP^+^ compartment reproducibly and almost exclusively exhibited a GC phenotype (Fig 8A, lower panels). Latently infected populations were also analyzed for cell surface markers consistent with a PC phenotype, which comprises ~20% of the CD3^-^YFP^+^ population (Fig 8B, upper panels). Although both reporter viruses were capable of efficiently establishing latency within the PC reservoir, we were consistently unable to detect M2 reporter activity in latently infected PCs using either the M2-mCherry or M2-Thy1.1 viruses (Fig 8B, lower panels).

**Fig 9:**
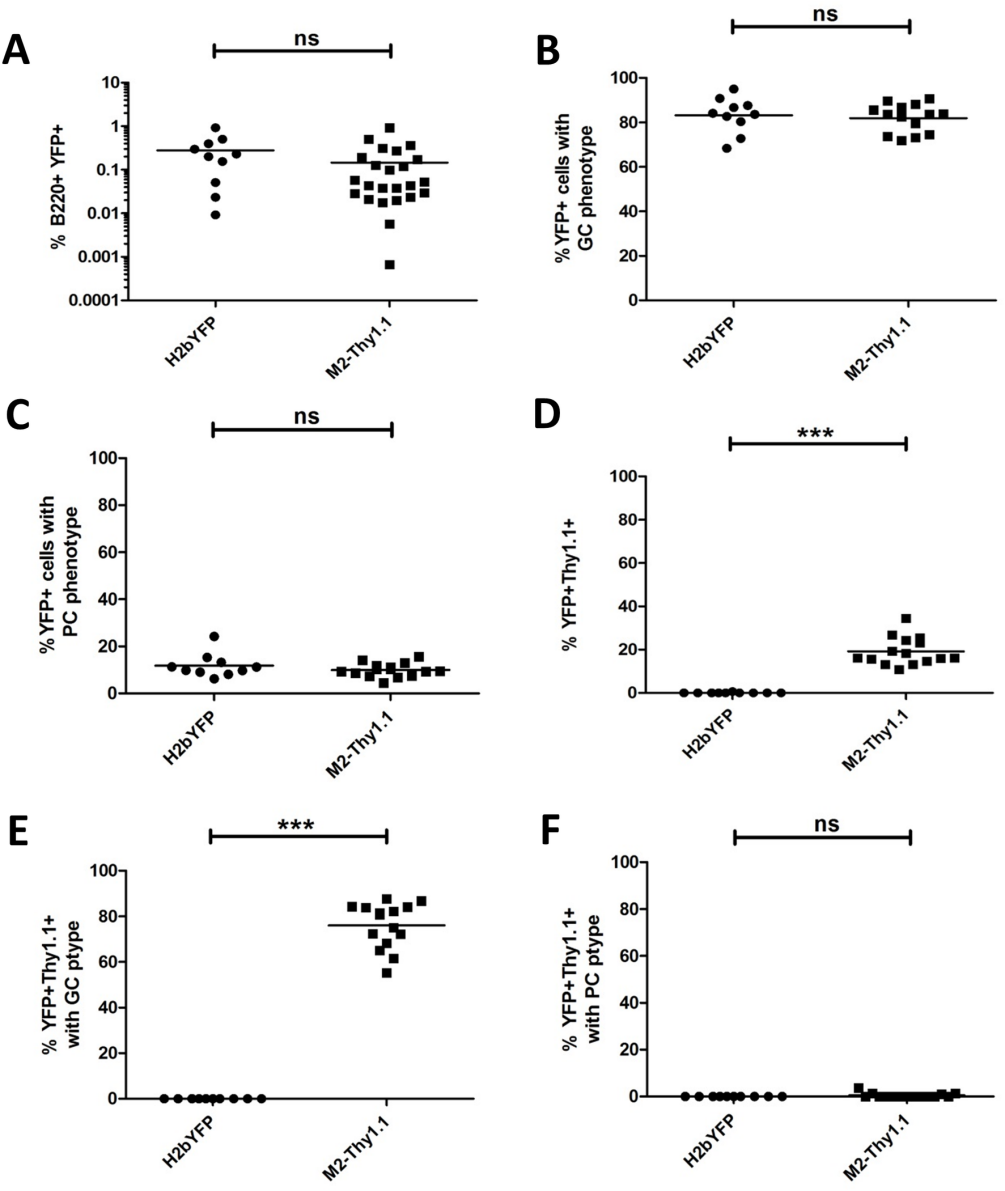
M2 expression is predominantly associated with a germinal center phenotype following intranasal infection. Mice were infected at 1000 PFU via the intranasal route with the parental MHV68 H2bYFP and MHV68 H2bYFP M2-Thy1.1 viruses. Splenocytes were harvested and cell surface markers were analyzed by flow cytometry at 16 dpi. Each data point represents one animal and the horizontal bar represents the mean. Statistics were determined by two-tailed unpaired t test with Welch’s correction. The frequency of B220^+^YFP^+^ latently infected B cells (A) exhibiting a germinal center (B) or plasma cell (C) phenotype were identified by flow cytometry as described in Fig 8. The B220^+^YFP^+^Thy1.1^+^ (M2 reporter) population was identified as described in Fig 7 and subphenotyped using surface markers consistent with a germinal center B cell (E) and plasma cell (F).

We subsequently evaluated MHV68 H2bYFP M2-Thy1.1 infection following inoculation at 1000 PFU IN, which represents a more stringent route of infection, in order to reveal any potential defects with viral trafficking to the spleen. Analysis of infected splenocytes at 16 dpi via the IN route demonstrated that the M2-Thy1.1 virus exhibited no significant alterations in latency establishment or viral trafficking to the GC and PC compartments (Fig 9A–9C). M2 reporter positive cells comprised on average ~20% of the B220^+^YFP^+^ population (Fig 9D), which was modestly reduced compared to the IP route (~40%; Fig 7B). Despite a slightly lower frequency of YFP^+^Thy1.1^+^ cells, we found that M2 reporter activity was reproducibly detected within the GC, but not the PC compartment (Fig 9E and 9F), which is consistent with the results obtained following IP infections (Fig 8). In conclusion, our M2 reporter viruses system has clearly established the latently infected GC B cell compartment as a critical site of potent M2 expression during latent MHV68 infection in vivo.

## Discussion

Prior studies have demonstrated a critical role for MHV68 M2 with respect to viral latency establishment and virus reactivation in a mouse model of infection [20–22]. In this study, we utilized complementary methods to further elucidate the context and potential impact of M2 expression with respect to B cell function in vivo. Our adoptive transfer studies are the first demonstration that M2 drives robust PC differentiation and IL10 production in vivo in the absence of other viral factors (Figs 3 and 4). However, we were unable to detect M2 reporter activity in the latently infected PC compartment (Figs 8 and 9), which may indicate that the M2 promoter is no longer active once the cell has reached a sufficient level of IRF4 expression required for terminal differentiation into a PC [43]. Additionally, the timing and low level of M2 protein expression that is required for PC differentiation, as demonstrated in our adoptive transfer system (Fig 4), may hinder our detection of M2 protein at 14 dpi in the M2 reporter virus system (Figs 8 and 9). Although we were unable to directly confirm protein expression in the latently infected PC, the adoptive transfer data fits into a well-established model in which M2-driven IRF4 expression facilitates PC differentiation during MHV68 infection [17, 20, 22, 44]. A significant portion of latently infected PCs can be generated via the extrafollicular pathway [45] and we have shown here that M2 drives robust PC differentiation in the absence of an ongoing GC reaction (Figs 3 and 4). How do latently infected PCs contribute to GHV pathogenesis? PC differentiation initiates lytic replication of EBV and KSHV via activation of viral transactivators by PC-specific transcription factors such as XBP-1 and Blimp-1 [23, 24, 46–48]. At least one study has reported that de novo KSHV infection drives human tonsillar B cells to proliferate and differentiate into plasmablasts that functionally and phenotypically resemble multicentric Castleman’s disease [49]. Therefore, PC differentiation represents a common aspect of GHV pathogenesis and this mechanism of virus reactivation has been proposed to facilitate virus transmission and maintenance of a stable life-long infection. For MHV68, PCs have been identified as the predominant source of infectious virus production, and virus reactivation is severely impaired in the absence of M2 [22]. Moreover, PCs appear to play an important role in virus trafficking and seeding of chronic MHV68 latency reservoirs. For example, an M2-null virus exhibits significantly impaired latency establishment in the spleen at 16 dpi, despite efficient viral replication in the lungs following low dose intranasal inoculation. Additionally, long term latency maintenance at 90 dpi was severely attenuated in mice lacking splenic PCs in a conditional Blimp-1 knockout model. In combination, our studies support a model in which GHVs play a direct role in driving PC differentiation which serves to facilitate reactivation of infectious virus and reseeding latency reservoirs within the infected host.

In addition to directly promoting virus reactivation, IL10 production by PCs has the potential to suppress humoral immunity and create a more permissive environment for viral infection. IL10 is a potent immunomodulatory cytokine that impairs T cell, macrophage and dendritic cell functions in a variety of infection settings (reviewed in [50]). GHVs exploit the IL10 signaling pathway by encoding viral IL10 homologs and/or enhancing IL10 expression from B cells, which promotes B cell expansion and abrogates immune recognition and subsequent eradication of infected B cells in vitro [19, 51–54]. During primary MHV68 infection M2 functions as an immunomodulatory molecule by elevating serum IL10 levels, attenuating antiviral CD8 T cell responses, and suppressing antigen-specific responses to MHV68 and subsequent challenges[19, 33]. Here we have shown that M2-driven PC differentiation is characterized by robust CD138 expression and IL10 production (Fig 4), which is consistent with M2-driven IRF4 production in B cells [17]. Interestingly, B cell IL10 production also attenuates aspects of innate and adaptive immunity in a salmonella infection model, and IRF4^Hi^CD138^Hi^ PCs have been identified as a potent source of IL10 [28, 29]. Therefore, we propose that latently infected PCs could serve as one potential source of immunsuppressive IL10 production during MHV68 infection. Moreover, dysregulated BCR signaling may represent a common mechanism by which GHVs and other pathogens promote regulatory PC generation as an immune evasion tactic during infection. The ultimate significance of IL10 expression in the context of GHV infection in vivo is still under debate, and investigations are currently underway to evaluate the contribution of host-derived IL10 to MHV68 pathogenesis.

The prevailing model of GHV pathogenesis requires that the virus traverse the GC compartment in order to gain access to the long-lived memory B cell compartment (reviewed in [55]). The GC reaction represents a competitive environment wherein B cells that do not receive rescue signals triggered by antigen recognition or T cell help are subjected to death by apoptosis while high affinity B cells that successfully compete for limited T cell help are positively selected to enter the long-lived B cell compartment [56, 57]. Similar to EBV, MHV68-latently infected B cells resemble, localize and participate in ongoing GC reactions. Importantly, and in contrast to EBV, T cell help is a demonstrated requirement for expansion of latently infected GC B cells and entry to the memory B cell pool, which serves as the long term latency reservoir for GHVs [15, 40, 58, 59]. M2 transcripts have been detected in GC B cells during chronic infection [5, 34] and our M2 reporter virus system has independently verified that the latently infected GC compartment is a site of robust M2 antigen expression at the peak of MHV68 latency, which was independent of the route of virus inoculation (Figs 8 and 9). In an increasingly hostile environment, M2 antigen expression could promote latently infected B cell survival and exit from the GC as a memory B cell. In this study, we show that M2 antigen expression in stimulated B cells was able to support, at least temporarily, the activated GC phenotype in the absence viral infection (Figs 3 and 4). Moreover, M2-driven signaling promotes the formation of B-T cell conjugates in the presence and absence of specific peptide [60], which in the context of the GC reaction could provide sufficient CD40 stimulation to enhance survival and selection of latently infected B into the memory B cell pool [61–63]. Further investigation is necessary in order to determine if M2 expression in GC B cells promotes viral trafficking to the memory B cell pool in vivo. Previous reports have confirmed the presence of M2 transcripts in memory B cells [5], but these analyses are inherently misleading and more sensitive and quantitative analyses are required to correlate MHV68 transcriptional programs with specific stages of B cell differentiation. Thus, enhanced characterizations of MHV68 latency antigen function and transcriptional programs may reveal common strategies by which GHVs effectively manipulate GC B cell biology to achieve short and/or long term persistence in vivo.

In conclusion, our studies have further validated a model in which M2 antigen expression dysregulates B cell activation, differentiation, and cytokine production to simultaneously thwart immune detection and eradication and promote MHV68 pathogenesis in the infected host. While the role of M2 expression within the GC B cell remains unknown, it has great potential to significantly influence both B and T cell responses to MHV68 infection. Therefore, our studies justify continued investigations that address the impact of M2 expression with respect to the global GC response during primary and secondary infections, as this may provide important insights with respect to GHV pathogenesis and associated disease.

## Materials and methods

### Ethics statement

This study was carried out in strict accordance with the recommendations in the Guide for the Care and Use of Laboratory Animals of the National Institutes of Health. The protocol was approved by the Emory University Institutional Animal Care and Use Committee (IACUC) and in accordance with established guidelines and policies at Emory University School of Medicine (protocol number: DAR 2003399-022419BN).

### Virus and tissue culture maintenance

BAC-derived MHV68 viruses were reconstituted following transfection of Vero-Cre cells, a generous gift provided by David Lieb [64]. Recombinant MHV68 viruses were propagated and titered on NIH3T12s (ATCC: CCL-164) as described previously [65]. Murine embryonic fibroblasts (MEFs) utilized in ex vivo reactivation assays were isolated from day 16 C57BL/6J embryos cultured as previously described [42]. Adherent cell lines were maintained in Dulbecco’s modification of Eagle medium (DMEM) supplemented with 10% fetal bovine serum, 2mM L-glutamine, and 100U penicillin and 100mg streptomycin per mL. Primary B cells isolated from C57BL/6J mice were maintained in Roswell Park Memorial Institute (RPMI) 1640 Medium supplemented with 10mM non-essential amino acids, 1mM sodium pyruvate and 10mM HEPES.

### Plasmids and retroviral constructs

Plasmid MSCV-M2-IRES-Thy1.1 (MSCV-M2) and MSCV-M2.stop-IRES-Thy1.1 (MSCV-M2.stop) have been previously described [19]. The mCherry protein sequence was fused to the C terminus of the M2 ORF in sequential steps as follows: the M2 sequence was amplified from MSCV-M2 using primers 5’-ctagagatctatggccccaacaccc-3’ and 5’-ctaggtttaaactctcctcgccccactc-3’ (flanking 5’ BglII and 3’ PmeI sites) and inserted into pCR-Blunt II Topo vector (Invitrogen) per manufacturer’s instructions; mCherry was amplified from pTREG-mCherry (Clontech) using primers 5’-ctaggtttaaacgtgagcaagggcgag-3’ and 5’-ctagctcgagagatcttcacttgtacagctcgtcc-3’ (flanking 5’ PmeI and 3’ Xho I BglII sites) and subsequently subcloned into to pCR-Blunt M2 vector following digestion with PmeI and XhoI. Lastly, a peptide sequence containing the AUI epitope (DTYRYI) and 30 amino acid F2A sequence [37] was inserted between the M2 ORF and the mCherry ORF using an overlapping PCR mutagenesis technique [66] with the following primer pairs: primers A&B 5’-ctagagatctatggccccaacaccc -3' and 5’-cgataggtatcctcctcgccccactcc -3'; primers C&D 5’-ggcgaggaggatacctatcgctatatacacaagcaaaagatcgttgcaccagttaagcaga ctctgaattttgacc-3' and 5’-cgtttaaactgggcccagggttggactcaacgtctccggccaacttgagcaggtcaaaattca gagtctgc-3'; primers E&F 5’-cctgggcccagtttaaacgtgagcaagggc-3' and 5’-ctagagatcttcacttgtaca gctcgtccatg-3'. PCR products AB, CD, and EF were utilized in the second ligation PCR step to generate the final M2-AU1-F2A-mCherry transgene (M2-mCherry) that was subsequently subcloned into the MSCV-IRES-Thy1.1 using BglII sites as previously described [19] to generate MSCV-M2-mCherry.

### Retrovirus production, primary B cell isolation and transduction

Retroviruses were produced by transfecting retroviral packaging cell line BOSC23 (ATCC) with individual MSCV vectors as previously described [41]. Primary B cells were isolated from naïve C57BL6/J mouse spleens (8–12 weeks of age) by negative selection using the EasySep Mouse B cell Enrichment Kit (Stem Cell Technologies) per manufacturer’s instructions. Following overnight stimulation with LPS at 20-25ug/mL, primary B cells were transduced with retroviruses supplemented with 5ug/mL polybrene by spinoculation at 2500rpm for one hour at 30°C. Cells were analyzed by flow cytometry using three wells per condition at days 2–5 post-transduction. Supernatants were collected and stored at -80°C for subsequent analysis by ELISA. IL10 in primary B cell supernatants was measured using the BD OptEIA Mouse IL-10 ELISA Kit (BD biosciences) per manufacturer’s instructions.

### Flow cytometry

Blocking and detection antibodies were diluted in PBS supplemented with 2% FBS and 1mM EDTA. Splenocytes were blocked with anti-CD16/32 (BD bioscience) for 15 minutes on ice prior to surface staining for 30 minutes on ice. Antibodies used in this study: B220-Pac Blue, CD138-BV650 and -APC, CD45.1-FITC, CD3-PerCp, CD4-PerCp, CD8-PerCp, Thy1.1-PE, CD95-PE/Cy7, GL7-APC, and CD19-Pac Blue, -BV650, -FITC,- PerCp, -PE, -PE/Cy7, -APC, -APC/Cy7, -Alexa Fuor 594 (BD bioscience, eBioscience, or Biolegend). For intracellular cytokine staining, unstimulated cells were fixed with 4% paraformaldehyde/PBS solution after surface staining step. Cells were subsequently permeabilized using the BD Cytofix/Cytoperm Fixation/Permeabilization Kit (BD biosciences) per manufacturer’s instructions prior to staining with IL10-PE/Cy7 (Biolegend). Dead cells were labeled with fixable viability dye eFluor780 (eBioscience) per manufacturer’s instructions. Cells were analyzed on a BD LSRII flow cytometer and data was analyzed using FlowJo software.

### Construction of recombinant viruses

M2-mCherry H2bYFP and M2-Thy1.1 H2bYFP bacterial artificial clones (BACs) were generated utilizing the galK selection method [39]. The M2 locus in the background of the previously described recombinant MHV68-H2bYFP genome [38] was replaced with galK gene as previously described [41]. Briefly, the galK cassette was amplified with primers flanked with 50bp sequence homology to the target sequence (5’-aggcgtgtttaaagaaaaagttatgttctgcgtta gcaccttcactgttacctgttgacaattaatcatcggca-3’ and 5’-agggggtttcaacaggcactagtctgatgaggtttcgtttt caggtaatgtcagcactgtcctgctcctt-3’) prior to electroporation of SW102 cells harboring the MHV68 H2bYFP BAC. The M2/galK intermediates exhibiting galactokinase activity were selected as previously described [41] and the presence of the desired insertion at the M2 locus was confirmed by restriction fragment length polymorphism (RFLP) analysis. M2-mCherry and M2-Thy1.1 sequences were amplified from the MSCV-M2-mCherry and MSCV-M2 vectors, respectively, with primers containing the 50bp homology arms and each cassette was electroporated into SW102 cells harboring the M2/galK intermediate. Positive recombinants were identified by PCR colony screen for the presence of the desired sequence and further evaluated by sequencing and RFLP analysis.

### Mice, infection, and adoptive transfer

For experimental infections, female C57BL6/J mice at 6–8 weeks of age were purchased from Jackson labs (Bar Harbor, ME) and were infected between 8–12 weeks of age. Mice were housed and maintained at the Whitehead vivarium according to Emory University and IACUC guidelines. Mice were anesthetized with isoflourane prior to infection via the intranasal or intraperitoneal route with 1000PFU of the MHV68 viruses. Mice were sacrificed at the indicated timepoints by CO_2_ inhalation per AVMA guidelines and spleens were harvested and processed as described previously [38]. Splenocytes from individual mice were analyzed by flow cytometry and splenocytes from 4–5 mice per experimental group were pooled for ex vivo virus reactivation analyses. For adoptive transfer studies, female B6.SJL-*Ptprc*^*a*^ *Pepc*^*b*^/BoyJ mice (Jackson labs) were used as donors for primary B cell isolation and retroviral transduction as described above. At one day post-transduction,10^7^ mock or transduced B cells were adoptively transferred into the peritoneum of naïve C57BL/6J mice at 8–12 weeks of age. Splenocytes were harvested from adoptive transfer recipients (3–4 mice/experimental group) at one and five days post transfer and analyzed by flow cytometry.

### Limiting dilution ex vivo reactivation analysis

Reactivation of infectious virus from latently infected splenocytes was evaluated by utilizing a limiting dilution ex-vivo reactivation assay as previously described [67]. Briefly, infected splenocytes were pooled from 3–5 mice per condition and plated in 12 two-fold serial dilutions onto MEF monolayers in 96-well tissue culture plates. Cytopathic effect was scored for each well (24 wells/dilution) at 14–21 days post-explant.

### Statistical analysis

GraphPad Prism software (San Diego, CA) was used to generate data graphs and perform statistical analyses. For line and bar graphs, the mean and standard deviation were plotted for each condition in triplicate. For scatter plots, each data point represents on animal and the horizontal bar represents the mean. Statistical significance between two conditions was determined by two-tailed unpaired t test with Welch’s correction. For three or more conditions, statistical significance was evaluated by one way analysis of variance (one way ANOVA) analysis followed by Bonferonni’s multiple comparisons post-tests. For reporting of absolute numbers of B cell populations in adoptive transfer recipients, 1 cell was added to all values in order to avoid the undefined logarithm of zero.
